# Inhibiting intercrystalline reactions of anode with electrolytes for long-cycling lithium batteries

**DOI:** 10.1126/sciadv.abq3445

**Published:** 2022-08-17

**Authors:** Peng Shi, Zhong-Heng Fu, Ming-Yue Zhou, Xiang Chen, Nan Yao, Li-Peng Hou, Chen-Zi Zhao, Bo-Quan Li, Jia-Qi Huang, Xue-Qiang Zhang, Qiang Zhang

**Affiliations:** ^1^ Beijing Key Laboratory of Green Chemical Reaction Engineering and Technology, Department of Chemical Engineering, Tsinghua University, Beijing 100084, China.; ^2^ State Key Laboratory of Automotive Safety and Energy, School of Vehicle and Mobility, Tsinghua University, Beijing 100084, P. R. China.; ^3^ Advanced Research Institute for Multidisciplinary Science, Beijing Institute of Technology, Beijing 100081, P. R. China.; ^4^ School of Materials Science and Engineering, Beijing Institute of Technology, Beijing 100081, P. R. China.

## Abstract

The life span of lithium batteries as energy storage devices is plagued by irreversible interfacial reactions between reactive anodes and electrolytes. Occurring on polycrystal surface, the reaction process is inevitably affected by the surface microstructure of anodes, of which the understanding is imperative but rarely touched. Here, the effect of grain boundary of lithium metal anodes on the reactions was investigated. The reactions preferentially occur at the grain boundary, resulting in intercrystalline reactions. An aluminum (Al)–based heteroatom-concentrated grain boundary (Al-HCGB), where Al atoms concentrate at grain boundary, was designed to inhibit the intercrystalline reactions. In particular, the scalable preparation of Al-HCGB was demonstrated, with which the cycling performance of a pouch cell (355 Wh kg^−1^) was significantly improved. This work opens a new avenue to explore the effect of the surface microstructure of anodes on the interfacial reaction process and provides an effective strategy to inhibit reactions between anodes and electrolytes for long–life-span practical lithium batteries.

## Teaser

A heteroatom-concentrated grain boundary was proposed to inhibit intercrystalline reactions for stable lithium batteries.

## INTRODUCTION

High–energy density and long-cycling lithium (Li) batteries as energy storage devices are indispensable in pursuit of nonfossil and wireless society (*1*–*3*). For nonaqueous battery system, a high–specific capacity anode with a low electrode potential (generally <0.5 V versus Li/Li^+^), such as graphite, silicon, and Li, is necessary to maximize specific energy of batteries (*4*–*7*). However, irreversible reactions inevitably occur between highly reactive anodes and electrolytes, which severely plagues the life span of batteries (*8*–*10*). Despite the formation of solid electrolyte interphase (SEI), yet the irreversible reactions occur continuously because of the fragility of SEI (*11*–*13*). Therefore, inhibiting the reactions occurring at anode/electrolyte interface is necessary for long-cycling rechargeable batteries (*14*–*17*).

To inhibit the reactions, tremendous efforts have been devoted to regulating anode/electrolyte interface from the scope of electrolytes, such as regulating electrolyte formulations to form stable SEI and designing artificial coating to block the contact between anodes and electrolytes (*18*–*27*). However, the effect of the surface structure of anodes on inhibiting reactions is rarely touched despite its critical role.

The reactions between anodes and electrolytes occur on the surface of anodes intrinsically. Thus, the effect of the surface structure of anodes on the reaction process should be equally emphasized in addition to the high reactivity of anodes thermodynamically. From a microcosmic view, an anode is generally composed of massive crystalline grains instead of a single crystal (*28*, *29*). Abundant grain boundaries (GBs) are exposed to electrolytes in addition to crystal planes. In general, GB has higher reactivity than the crystal plane (*30*, *31*). The reactions are inclined to occur at GBs preferentially and then spread along GBs into the inside, that is, intercrystalline reactions. Therefore, understanding the reaction process of electrolytes on the surface of anodes at a microcosmic level is urgent, and modifying GBs emerges as a fresh strategy to inhibit the intercrystalline reactions.

**Fig. 1.**
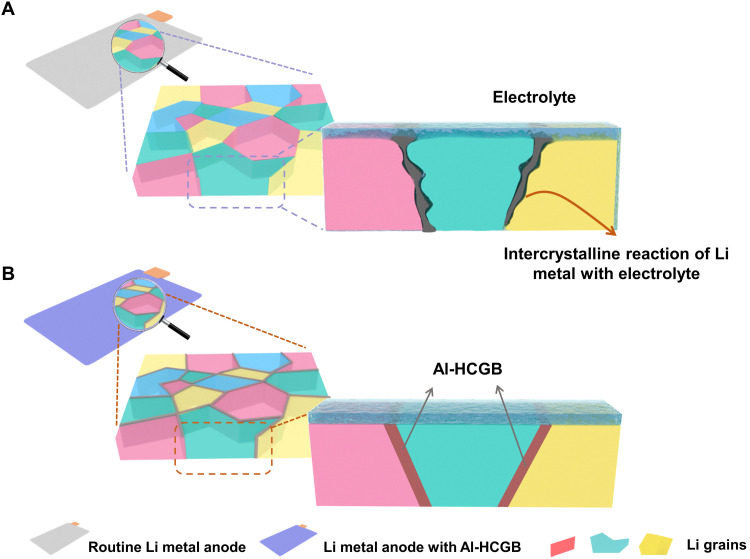
Scheme of the process of intercrystalline reaction of Li metal with electrolytes. (**A**) A routine Li metal and (**B**) Li metal with Al-HCGB. Intercrystalline reactions of Li metal preferentially occur at the GB and cause the consumption of Li metal and electrolytes. In contrast, Al-HCGB-Li can inhibit intercrystalline reactions and render a stable Li metal anode.

In this contribution, the effect of GB of anodes on the reaction process between anodes and electrolytes was disclosed. Li metal anode was used as a typical anode owing to its unique ability to construct high–energy density batteries (over 350 Wh kg^−1^ at a cell level). The reactions are inclined to occur at GBs preferentially, because the Li at GB has higher energy than the Li at the crystal plane (Fig. 1A). To inhibit the intercrystalline reactions, we proposed modifying GBs of Li metal anodes by constructing aluminum (Al)–based heteroatom-concentrated grain boundary (Al-HCGB; Fig. 1B), where Al atoms concentrate at GBs without forming a detectable phase. At Al-HCGB, electron transfers from Li to Al atoms and increases the reaction resistance between Li metal and electrolytes, which inhibits the intercrystalline reactions and mitigates the loss of active Li and electrolytes. A battery with a Li metal anode containing Al-HCGB exhibits twice the life span compared with a routine Li metal anode in various types of electrolytes. In particular, the scalable preparation of Al-HCGB was demonstrated, with which a pouch cell of 355 Wh kg^−1^ maintains 86% retention of initial capacity after 113 cycles.

## RESULTS

### The origin of intercrystalline reactions of Li metal anodes

**Fig. 2.**
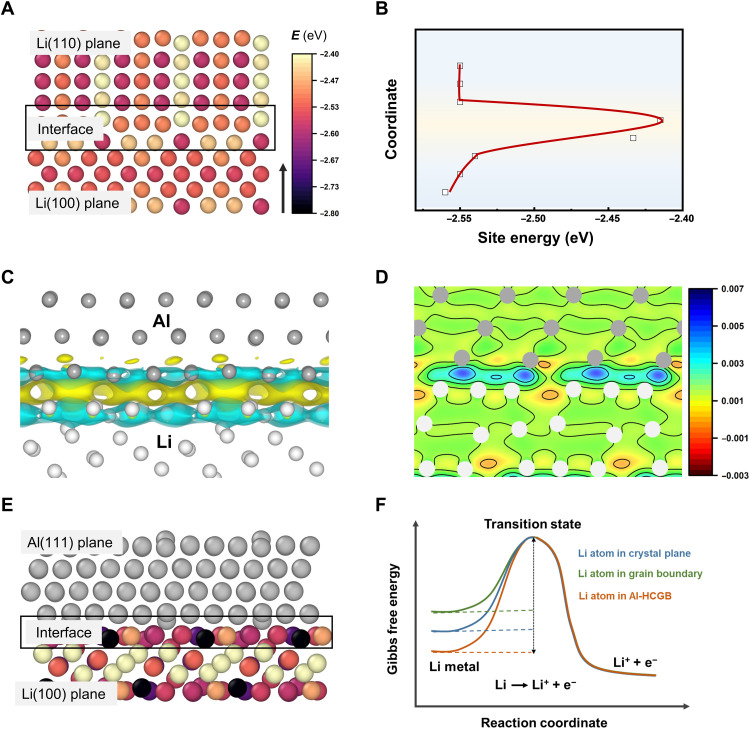
Mechanism of inhibiting intercrystalline reactions by Al-HCGB. (**A**) Site energy (*E*_site_) of Li atoms in Li(110), Li(100), and their interface. The color bar of the site energy is shown on the right. (**B**) Evolution of *E*_site_ along with the perpendicular to surface [the direction of the arrow in (A)]. (**C**) Deformation charge density distribution at the interface between Li and Al. Li and Al atoms are colored as white and gray, respectively. Yellow and cyan color indicate an accumulation and depletion of the electron, respectively (isosurfaces: 0.001 |e|/*a*_0_^3^, where *a*_0_ is Bohr radius). (**D**) Corresponding two-dimensional (2D) section of (C). (**E**) Site energy of the Li atom in Li(100) plane and the interface between Li and Al metals. The color bar is the same as that of (A). (**F**) Scheme of Gibbs free energy change for the reaction of Li − e^−^ = Li^+^ at crystal plane, GB, and Al-HCGB.

Li metal batteries generally use a Li foil as an anode. From a microcosmic view, a Li foil is composed of massive crystalline grains rather than a single crystal. There are various types of GBs and crystal planes on the surface of a common Li foil, which is referred to as the surface microstructure of a Li foil. Li metal from either crystal planes or GBs can react with electrolytes. To quantitatively describe the reactivity of Li atoms at crystal planes and GBs, site energy (*E*_site_) was determined by first-principles calculations based on density functional theory (DFT) (*32*). *E*_site_ is defined as *E*_site_ = *E*_total_ − *E*_vacancy_, where *E*_total_ and *E*_vacancy_ are the total energy of the pristine defect-free model and the model containing a Li vacancy, respectively. Accordingly, a Li atom with a high *E*_site_ exhibits a high reactivity. A close average *E*_site_ between Li(100) and Li(110) planes (difference within 0.02 eV) indicates a similar reactivity of Li on the two crystallographic planes (Fig. 2, A and B, and fig. S1). However, the average *E*_site_ of Li atoms at the GB between Li(100) and Li(110) planes is significantly higher than that in crystal planes (−2.41 versus −2.55 eV), implying that Li at GBs exhibits a higher reactivity. Consequently, the reactions between Li and electrolyte are inclined to start at GBs and then spread along GBs, that is, intercrystalline reactions.

HCGB was proposed to inhibit the intercrystalline reactions of Li foil and electrolytes. When heteroatoms, such as Al atoms, contact with Li atoms, the electrons transfer from Li to Al atoms because the electronegativity of Al atoms is stronger than that of Li atoms, which is confirmed by deformation charge density analyses (Fig. 2, C and D). The charge transfer reduces the electron density around Li atoms and further decreases the reactivity of Li. Therefore, the average *E*_site_ of Li atoms at GBs decreases from −2.41 to −2.60 eV when Al atoms are introduced to the GB (Fig. 2E). Similar to Al atoms, the introduction of magnesium (Mg) atoms at the GB delivers an average *E*_site_ of −2.51 eV (fig. S2). Therefore, it is a reasonable route to decrease the reactivity of Li atoms at GBs by introducing heteroatoms at GBs (fig. S3). With the decrease of *E*_site_, the energy barrier of the reaction of Li − e^−^ = Li^+^ is supposed to be increased (Fig. 2F) (*33*). Therefore, Al-HCGB can inhibit intercrystalline reactions of Li metal with electrolytes compared with routine GBs, and thus, the consumption rate of active Li and electrolytes is prominently mitigated.

### Construction of Al-HCGB

**Fig. 3.**
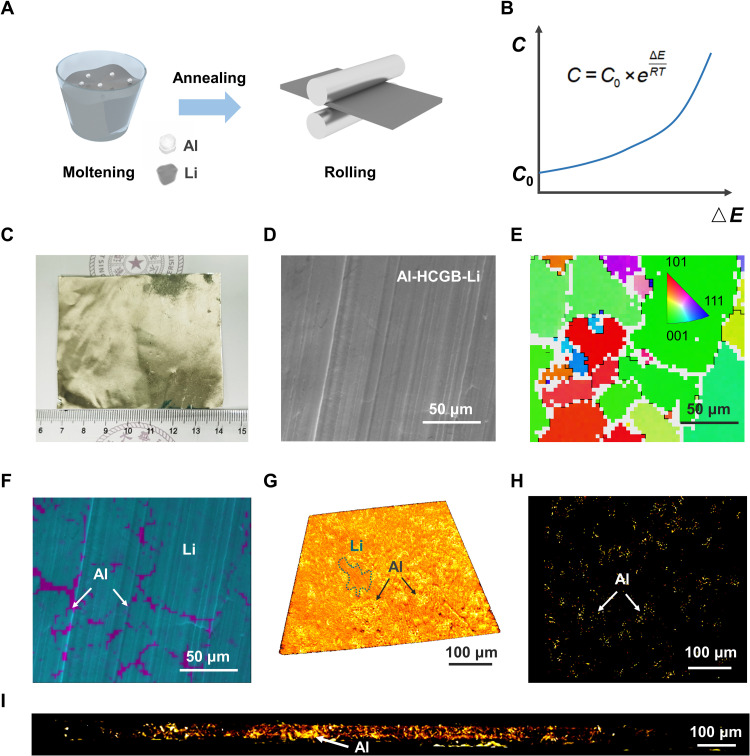
Construction of Al-HCGB. (**A**) The fabrication process of Al-HCGB-Li. (**B**) Relation of the concentration of Al atoms at GBs and the distortion energy. ∆*E* = *E*_interior_ − *E*_boundary_, which represents the difference between the distortion energy of the atom at its current location and that of the atom at the GB. *C*_0_ represents the concentration of Al atoms inside the grain. (**C**) Optical image of Al-HCGB-Li foil. (**D**) SEM image of Al-HCGB-Li. (**E**) EBSD mapping and the inverse pole figure coloring maps of Al-HCGB-Li. (**F**) EDS mapping of Al element in Al-HCGB-Li. Li and Al elements were colored as cyan and purple, respectively. (**G**) Distribution of Al inside Al-HCGB-Li by XRM. (**H**) Transverse and (**I**) longitudinal sectional views of (G). Al element is highlighted by adjusting the contrast of the images.

To fabricate a Li metal anode with Al-HCGB (Al-HCGB-Li), Li metal is molten first, and then a small amount of Al metal is added (Fig. 3A and fig. S4). The mass ratio of Li and Al metal is 95:5. The Al-HCGB-Li anode delivers a large specific capacity as high as 3496 mAh g^−1^ based on the weight of a whole anode (fig. S5). There are no intermetallic compounds detected when the mass ratio of Al is less than 5 weight %, which can be confirmed by the single peak of Li(200) in the x-ray diffraction (XRD) pattern of Al-HCGB-Li (figs. S6 and S7) (*34*–*36*). When heteroatoms are introduced into the grain, they tend to distribute in locations where the distortion energy can be reduced during the process of annealing. Therefore, Al atoms spontaneously concentrate at GBs to minimize the distortion energy of the whole system, which has been named as GB segregation (Fig. 3B) (*37*, *38*). The relationship of the concentration of Al atoms at GBs (*C*) and the gap of distortion energy between the boundary (*E*_boundary_) and interior (*E*_interior_) satisfies the Arrhenius equation

$$C=C_{0}\times e^{\frac{\left(E_{\text{interior}}-E_{\text{boundary}}\right)}{\mathrm{RT}}}$$

where *C*_0_ represents the concentration of Al atom inside the grain, *R* is the gas constant, and *T* is the temperature.

The annealed Al-HCGB-Li was rolled into a Li foil with a thickness of 50 μm for characterization and battery assembly, and the scalable preparation of Al-HCGB-Li was feasible (Fig. 3C and fig. S8). Scanning electron microscopy–electron backscatter diffraction–energy-dispersive spectroscopy (SEM-EBSD-EDS) was used to investigate the morphology of Al-HCGB-Li foil and the distribution of Al atoms in Al-HCGB-Li (*39*). The surface of Al-HCGB-Li foils is flat and smooth (Fig. 3D). The band contrast image, EBSD mapping, and the inverse pole figure coloring map obtained from the same region exhibit that the Al-HCGB-Li is composed of multiple grains with different orientations, which are represented by various colors (Fig. 3E and fig. S9) (*28*). EDS mapping of Al element on the same region demonstrates that most Al atoms concentrate at GBs (Fig. 3F). Furthermore, a three-dimensional x-ray microscope (3D XRM) is used to probe the distribution of Al inside Al-HCGB-Li (Fig. 3G) (*40*–*42*). Li and Al elements can be unambiguously distinguished because of the different contrasts in the 3D XRM image. The color of Li is darker than Al because Al has a larger atomic mass. From the cross-sectional view, bright spots of Al suggest a state of segregation instead of uniform distribution at an atomic scale inside Al-HCGB-Li, demonstrating that Al atoms concentrate at GBs both on the surface and inside (Fig. 3, H and I). Therefore, Al-HCGB is successfully constructed in a Li foil, and Al-HCGB-Li is obtained to evaluate the effect of Al-HCGB in inhibiting the intercrystalline reactions.

### Inhibiting intercrystalline reactions by Al-HCGB

The air with a humidity of 34%, which has higher reactivity than electrolytes, was first used to evaluate the effect of Al-HCGB in inhibiting intercrystalline reactions at GBs. Routine Li was fabricated by the molten method, as described in the Supplementary Materials, to exclude the effect of the fabrication process on the stability of Li metal anodes. When routine Li and Al-HCGB-Li are exposed to the air after 48 hours, the mass of routine Li increases more than 110% because of the intercrystalline reactions induced by air, which is much larger than that of Al-HCGB-Li (60%). In addition, the metallic luster of a routine Li completely fades after 24 hours, but Al-HCGB-Li is still shiny after 24 hours, implying the substantially inhibited intercrystalline reactions on Al-HCGB-Li (fig. S10).

**Fig. 4.**
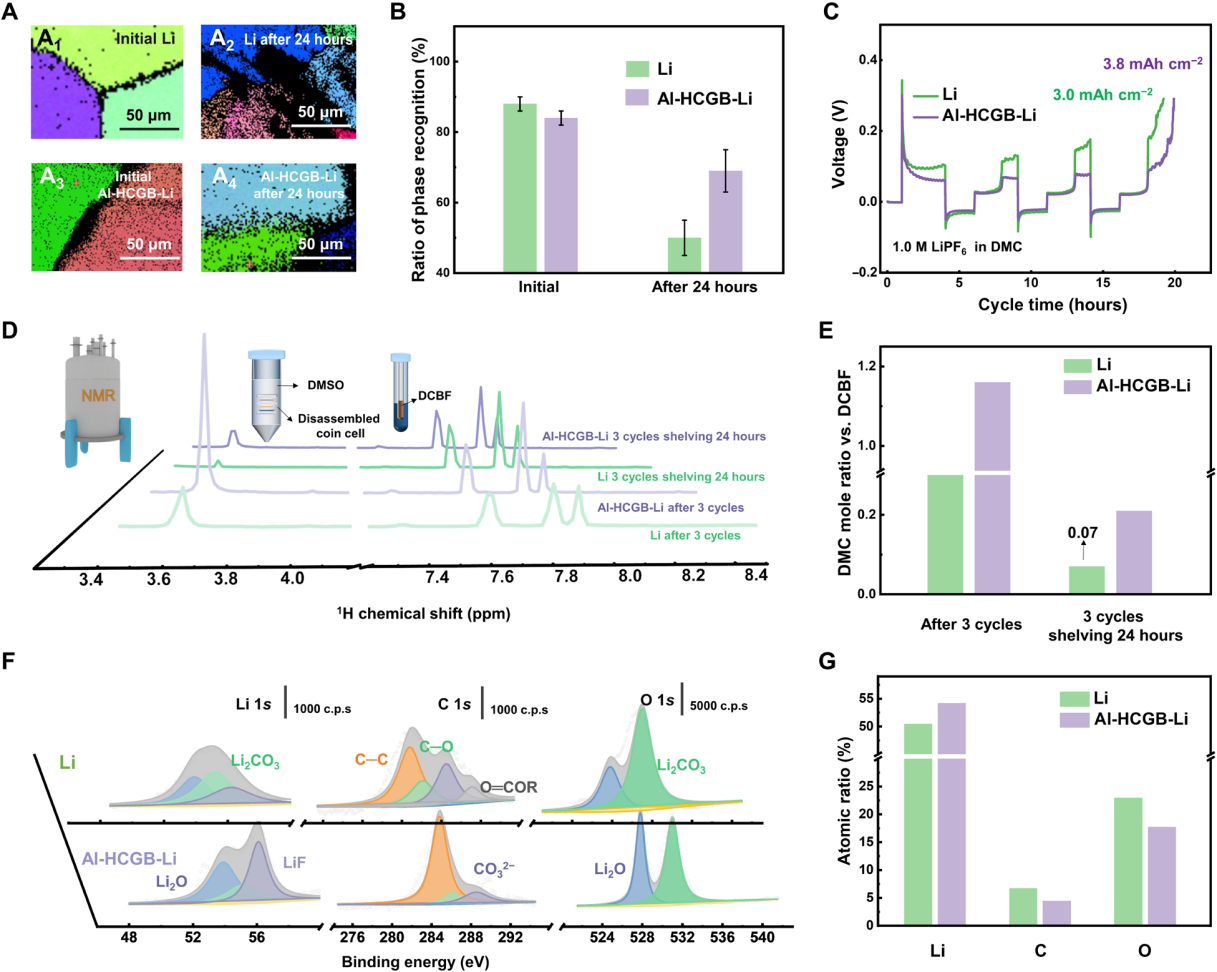
Inhibiting intercrystalline reactions by Al-HCGB. (**A**) EBSD mapping of (A_1_ and A_2_) routine Li and (A_3_ and A_4_) Al-HCGB-Li foil at (A_1_ and A_3_) initial state and (A_2_ and A_4_) soaked in electrolytes after 24 hours. Different colors represent different orientations. The black part represents the unrecognizable phase that can be regarded as reaction products. (**B**) The ratio of phase recognition in (A) according to image recognition. Repeated experiments provide error bars for phase recognition in figs. S10 and S11. (**C**) Li stripping capacity of routine Li and Al-HCGB-Li after 3 cycles. (**D**) ^1^H-NMR spectra of the electrolyte from the cells with routine Li and Al-HCGB-Li metal after 3 cycles and shelving for 24 hours. DMSO, dimethyl sulfoxide. (**E**) Molar ratio of DMC calculated according to (D). (**F**) XPS spectra of SEI on routine Li and Al-HCGB-Li anodes. c.p.s., counts per second. (**G**) The atomic ratio of Li, C, and O on the surface of routine Li and Al-HCGB-Li.

Al-HCGB-Li and routine Li were soaked in an aggressive electrolyte [1.0 M lithium hexafluorophosphate (LiPF_6_) dissolved in dimethyl carbonate (DMC)] to evaluate the effect of Al-HCGB on inhibiting the intercrystalline reactions of Li with electrolyte in batteries. The surface of routine Li is coarse, while the surface of Al-HCGB-Li is smooth after soaking for 24 hours (fig. S11). EBSD mappings were further used to quantitatively monitor the reaction process of routine Li and Al-HCGB-Li (Fig. 4A and figs. S12 and S13). The scanning pattern with discernible orientations is identified as active Li, whereas the black part represents the unrecognizable phase that can be regarded as reaction products, such as Li_2_O and Li_2_CO_3_. The GBs of routine Li and Al-HCGB-Li are clear at pristine state. Afterward, intercrystalline reactions take place at GBs, which extends along GBs. After 24 hours, routine Li is covered by an unrecognized phase with a larger area compared with Al-HCGB-Li. The area of the recognizable phase was quantified on the basis of image recognition. The ratio of recognizable phase decreases from 88 to 50% after 24 hours for routine Li (Fig. 4B). However, the recognizable phase of Al-HCGB-Li only reduces from 84 to 69%. Therefore, the intercrystalline reactions of Li with electrolytes on Al-HCGB-Li are substantially inhibited.

The residual content of active Li metal and electrolyte components was measured quantificationally to probe the mitigated reactions on Al-HCGB-Li. The half-cell with routine Li as a counter electrode, namely, routine Li (anode) | routine Li (cathode) or routine Li (anode) | Al-HCGB-Li (cathode) cell, was used. The thickness of the electrode was 50 μm. Li (3.0 mAh cm^−2^) was first stripped from the cathode, and then 2.0 mAh cm^−2^ of Li was plated on the cathode for exposing more active Li on the cathode and accelerating the consumption rate of active Li. After consecutive asymmetric cycling for 3 cycles, active Li of 3.8 mAh cm^−2^ remains in Al-HCGB-Li, which is larger than that of 3.0 mAh cm^−2^ in routine Li, indicating that the consumption content of active Li is significantly reduced by 11% (Fig. 4C). After cycling, the above cells were disassembled for electrolyte extraction, and liquid nuclear magnetic resonance of ^1^H (^1^H-NMR) was used to determine the consumption content of solvents (fig. S14) (*43*–*45*). The peak at 3.6 parts per million (ppm) in ^1^H-NMR is assigned to the H from DMC, and the peaks after 7.0 ppm belong to the internal standard substance of 2,4-dichlorobenzotrifluoride (DCBF; Fig. 4D and fig. S14). The content of DCBF is fixed and is served as the benchmark. As shown in Fig. 4E, the molar ratio between DMC and DCBF is 1.16 in the cell with an Al-HCGB-Li anode after 3 cycles, which is higher than that with a routine Li anode (0.34), indicating that the consumption content of DMC solvent in Al-HCGB-Li has been reduced as one-third of that in routine Li. To verify the reactions between electrolytes and Li metal during storage, we set the disassembled cell in a sealed tube for 24 hours, and thus, the cycled anode had sufficient reaction time with organic electrolytes. The remaining content of DMC in the cell with an Al-HCGB-Li anode is three times larger than that with a routine Li anode after shelving for 24 hours. In addition, the electrolyte of 1.0 M LiPF_6_ in DMC/fluoroethylene carbonate (FEC; the volume ratio is 4:1) was used to the universality of the mitigated reactions. The changes of FEC and PF_6_^−^ at 0 cycles is set as 0.00% for comparison (table S1). According to the results of ^1^H-NMR, the change of FEC solvent of routine Li is nearly three times than that of Al-HCGB-Li after 10 cycles, and the change of PF_6_^−^ of routine Li is more than twice than that of Al-HCGB-Li, which indicates that the Al-HCGB can work in the different electrolytes and after long cycles. The components of SEI on a routine Li and an Al-HCGB-Li were further investigated to confirm the decreased consumption content of electrolytes (Fig. 4, F and G). SEI on a routine Li and an Al-HCGB-Li share similar components, such as LiF and Li_2_O (*16*). However, the SEI on routine Li has a higher atomic ratio of C and O than that on Al-HCGB-Li, implying that the decomposition of solvents on Al-HCGB-Li is mitigated during cycling, which is consistent with the result of the decreased consumption content of DMC. Consequently, the consumption rate of active Li and electrolytes is significantly reduced because of the mitigated intercrystalline reactions of Li metal with electrolytes induced by Al-HCGB. In addition, the state of Al atoms before and after cycling was detected by x-ray photoelectron spectroscopy (XPS; fig. S15). The proportion of Al-containing species has little changes, indicating that the state of Al atoms is stable during cycling.

### Stability of Al-HCGB-Li in Li metal pouch cells

In practical Li metal batteries, excessive Li at the anode side (i.e., 1 < N/P ratio < 2.5, N/P ratio is the capacity ratio between negative and positive electrode), denoted as bulk Li to differentiate from the deposited Li from cathode side, is generally used to avoid rapid depletion of active Li (fig. S16A) (*46*). Bulk Li has to be stripped to achieve a required stripping capacity because the stripping capacity of deposited Li is not enough owing to the low Coulombic efficiency (CE; fig. S16B) (*47*, *48*). When bulk Li is stripping, bulk Li reacts with electrolytes due to the crack of existing SEI. In particular, the amount of exposed bulk Li increases with a decreasing CE and an increasing capacity of deposited Li, aggravating the reactions and inducing the rapid depletion of bulk Li at a low N/P ratio. Consequently, using bulk Li with Al-HCGB emerges as a feasible and effective solution to inhibit irreversible reactions in Li metal batteries.

**Fig. 5.**
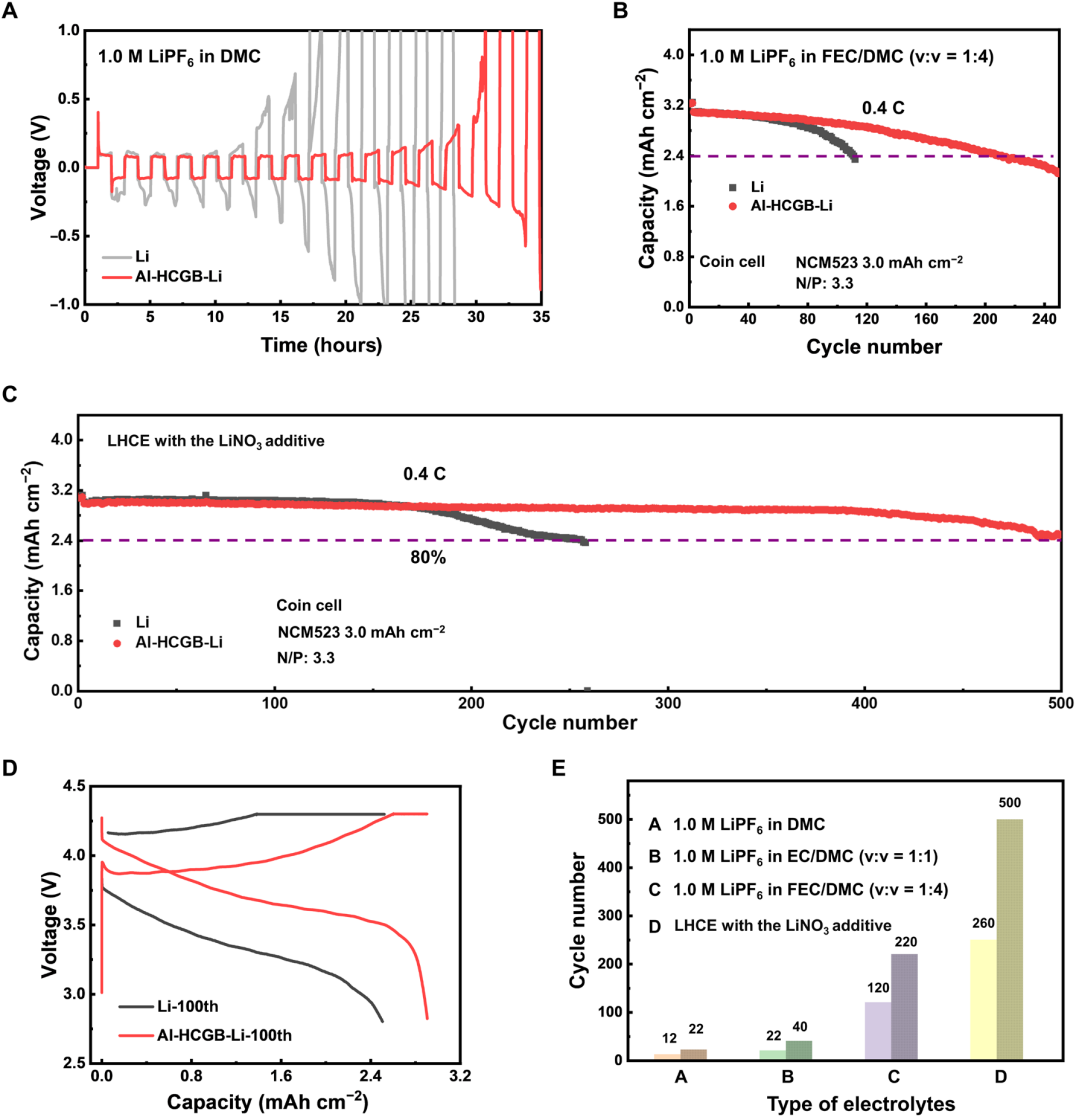
Stability of Al-HCGB-Li in practical Li metal batteries. (**A**) Voltage profiles of routine Li | Li and Al-HCGB-Li | Al-HCGB-Li cells with a capacity of 1.0 mAh cm^−2^ at a current density of 1.0 mA cm^−2^ in the electrolyte of 1.0 M LiPF_6_ in EC/DMC (v:v = 1:1). Cycling performance of Li | NCM523 batteries at 0.4 C in the electrolyte of (**B**) 1.0 M LiPF_6_ in FEC/DMC and (**C**) LHCEs with LiNO_3_ additives. (**D**) Corresponding voltage profiles of coin cells at the 100th cycle with FEC-containing electrolytes. (**E**) Summary of the cycling performance of coin cells with different electrolyte systems.

Li | Li symmetrical batteries are adopted to investigate the stability of Al-HCGB-Li in comparison to routine Li. DMC, which is an unstable solvent to Li metal, is selected to prepare the electrolyte (1.0 M LiPF_6_ in DMC) with high reactivity to amplify the difference in reaction resistance (*49*). A lower voltage hysteresis and longer cycles are achieved in the cells with Al-HCGB-Li compared with routine Li (Fig. 5A). The cell with routine Li exhibits a gradually increasing hysteresis to 500 mV within 15 hours, while the cell with Al-HCGB-Li maintains a much smaller hysteresis of 200 mV after 25 hours. The sharp increase in polarization of routine Li is attributed to the rapid accumulation of dead Li, as indicated by the rough surface of routine Li after 5 cycles (fig. S17). In contrast, the surface of Al-HCGB-Li is smooth. Obvious bulk active Li can be observed in Al-HCGB-Li after 5 cycles, but routine Li has been completely pulverized (fig. S18).

Al-HCGB-Li was further evaluated in full batteries under practical conditions. The full battery was assembled with a high-loading LiNi_0.5_Co_0.2_Mn_0.3_O_2_ (NCM523, 3.0 mAh cm^−2^) cathode and a low N/P ratio (3.3). The full batteries with routine Li and Al-HCGB-Li perform 12 and 22 cycles (fig. S19), respectively, in the electrolyte of 1.0 M LiPF_6_ in DMC, demonstrating that the stability of Al-HCGB-Li is improved owing to the inhibited intercrystalline reactions. Furthermore, the stability of Al-HCGB-Li was evaluated in different electrolyte systems to confirm the generality of this strategy (Fig. 5, B and E). In the electrolyte of 1.0 M LiPF_6_ in ethylene carbonate (EC)/DMC (the volume ratio is 1:1), the battery with routine Li rapidly fails after 22 cycles at 0.4 C under the benchmark of 80% capacity retention (fig. S20). By contrast, the cycle life of the battery with Al-HCGB-Li is prolonged to 40 cycles. When using electrolyte systems with improved stability against Li, such as FEC-containing electrolytes and localized high concentration electrolytes (LHCEs), the cycle performance is remarkably enhanced by Al-HCGB-Li (*50*–*52*). In the electrolyte of 1.0 M LiPF_6_ in FEC/DMC (1:4, by volume), the battery with an Al-HCGB-Li delivers 220 cycles, which is a much extended life span compared with 120 cycles for routine Li (Fig. 5B). Moreover, Al-HCGB-Li | NCM523 battery performs 500 cycles at 0.4 C at 80% capacity retention, nearly twice as that with routine Li (260 cycles) in LHCEs with LiNO_3_ additives (Fig. 5C). The significantly increased cycle life demonstrates the high stability of Al-HCGB-Li in various electrolytes. Furthermore, the effect of Al-HCGB-Li on Li nucleation and plating is excluded. In the FEC-containing electrolyte, the morphology of Li deposition on the routine Li and Al-HCGB-Li anode is similar, which demonstrates that the Al-HCGB cannot directly regulate the behaviors of Li nucleation and plating (fig. S21). However, the surface of Al-HCGB-Li foil is relatively flat in comparison to routine Li metal after 5 cycles (fig. S22). From the cross-sectional SEM images, active Li can be observed in Al-HCGB-Li after 5 cycles, but routine Li has been partially pulverized (fig. S23). Merely dead Li can be observed from the surface of routine Li after 260 cycles; however, deposited Li can be identified from the surface of Al-HCGB-Li even after 500 cycles in LHCEs with LiNO_3_ additives (fig. S24). The middle voltage, as an indicator of polarization, is 3.59 V in the battery with Al-HCGB-Li, which is larger than 3.28 V of the battery with a routine Li at the 100th cycle in FEC-containing electrolytes (Fig. 5D). The improved middle voltage is also confirmed in LHCEs with LiNO_3_ additives (fig. S25). The stability of Al-HCGB-Li evaluated in different electrolyte systems is summarized in Fig. 5E, indicating that the intercrystalline reactions in Al-HCGB-Li are notably inhibited in various electrolytes. Al-HCGB decreases the consumption rate of active Li and electrolytes and mitigates the accumulation of dead Li. Therefore, the stability of a full battery with Al-HCGB-Li is remarkably improved under demanding conditions by inhibiting the intercrystalline reactions.

**Fig. 6.**
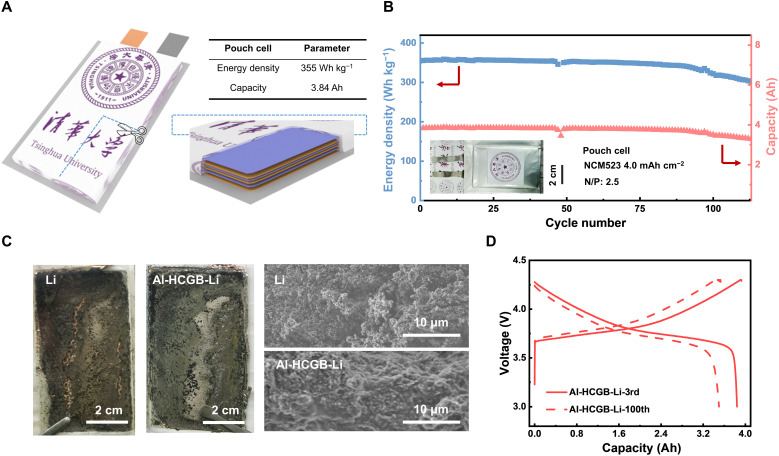
Pouch cells with Al-HCGB-Li. (**A**) Scheme of a pouch cell of 355 Wh kg^−1^ based on the mass of all components. (**B**) Cycling performance of a 3.84-Ah pouch cell with Al-HCGB-Li in the electrolyte of 1.0 M LiPF_6_ in FEC/DMC. (**C**) Photos and SEM images of routine Li and Al-HCGB-Li at a stripping state in pouch cells after 100 cycles. (**D**) Voltage capacity profiles of a pouch cell with Al-HCGB-Li anodes at the 3rd and 100th cycles.

The scalable preparation and cycle performance of Al-HCGB-Li were further demonstrated in a pouch cell with a high-loading NCM523 cathode of 4.0 mAh cm^−2^, lean electrolytes of 2.3 g Ah^−1^, and a low N/P ratio of 2.5 (table S2) (*51*, *53*, *54*). LiPF_6_ (1.0 M) in FEC/DMC (1:4, by volume) was selected as the electrolyte. The discharge capacity of the pouch cell is 3.84 Ah at the first cycle, and the specific density reaches up to 355 Wh kg^−1^ based on the mass of all components of a pouch cell (Fig. 6A). It can maintain 86% of the initial capacity after 113 cycles (Fig. 6B). The surface of routine Li at a stripping state is covered by chunks of dead Li after 100 cycles, and part of pulverized dead Li peels off from the Cu collector (Fig. 6C). In contrast, the morphology of Al-HCGB-Li is relatively flat and dense, and active Li can be observed from the surface of Al-HCGB-Li, indicating that Al-HCGB-Li can still participate in subsequent cycles. Meanwhile, the middle voltage only changed by 30 mV from the 3rd to 100th cycle because of the reduced accumulation of dead Li (Fig. 6D). Owing to the mitigated intercrystalline reactions, Al-HCGB-Li prominently reduces the consumption rate of active Li and thus contributes to improving cycle stability of the pouch cell, which is of great significance for practical applications.

At this stage, Al-HCGB has been confirmed as a reliable route to inhibit intercrystalline reactions of Li metal anodes experimentally and theoretically from a microcosmic view. Consequently, the surface microstructure of Li metal plays an essential role in dictating the reaction process. In addition to Al-HCGB, the feasibility of Mg-HCGB in inhibiting the reactions of electrolyte and bulk Li is also clarified (fig. S26). Thus, HCGB emerges as a primary but general strategy for intensive research. Furthermore, the proof of concept of HCGB in Li metal batteries provides a reference for other nonaqueous batteries with sodium, magnesium, silicon, and so on as anodes, because they are also polycrystalline microscopically. Therefore, HCGB affords an extended understanding and strategy in various nonaqueous batteries.

## DISCUSSION

In this contribution, the effect of GBs of Li metal anodes on the reactions between electrolytes and Li metal is disclosed. The reactions preferentially occur at GBs from a microcosmic view, that is, intercrystalline reactions. Al-HCGB, where Al concentrates at GBs on the surface of Li metal, is proposed to inhibit the intercrystalline reactions. Electrons transfer from Li to Al atom in Al-HCGB, which increases the energy barrier of reactions at GBs. Thus, the consumption rate of electrolyte and Li metal was prominently reduced because of the mitigated intercrystalline reactions. The Li metal battery with Al-HCGB-Li exhibited twice the life span compared with a routine Li in various electrolyte systems. A pouch cell of 355 Wh kg^−1^ underwent 113 cycles when it decreases to 86% of the initial capacity. This work provides a fresh understanding of the effect of surface microstructure on reactions at anode/electrolyte interface and proposes an effective and general strategy to inhibit intercrystalline reactions for long-cycling nonaqueous batteries.

## MATERIALS AND METHODS

### Fabrication of the Al-HCGB-Li foil

Li was first melted in a nickel crucible on a hot plate (C-MAG HP 4, IKA) at a temperature of 300°C in an Ar-filled glove box with O_2_ and H_2_O content below 0.1 ppm. Then, the Al metal (99%, Alfa) was immersed into molten Li to produce the Al-HCGB-Li electrode. The Li metal (99.9%, China Energy Lithium Co. Ltd.) and Al metal are at a mass ratio of 95:5. The solution of Al-HCGB-Li was maintained at 300°C for 30 min and naturally anneals to room temperature. The annealed Al-HCGB-Li was rolled to fabricate the electrode foil with a thickness of 50 μm. The rolling process was carried out in the dry room with a dew point temperature of −40°C.

### Material characterization

An SEM-EBSD-EDS combination system with the Aztec acquisition software (Oxford Instruments) was used to analyze the structure, crystal orientation, and phase of routine Li and Al-HCGB-Li foil. The surface of the sample was scraped by a blade until shiny.

An SEM (JSM 7401F, JEOL, Japan) and corresponding energy-dispersive elemental mapping were used to characterize the morphology of routine Li metal and Al-HCGB-Li foil. The electrode material samples obtained from disassembled cells were first cleaned by 1,2-dimethoxyethane (DME) solvent three times and then dried until the solvent was volatilized thoroughly in a glove box.

3D x-ray microscopy (ZEISS Xradia 510 Versa, Microscopy Customer Center Beijing) was used to get high-resolution 3D images. The volume resolution is 0.3 μm. XRD patterns were recorded on a Bruker D8 Avance diffractometer equipped with a Cu-K_α_ radiation source.

An Al K_α_ radiation (72 W, 12 kV) at a pressure of 10^−9^ torr was applied to obtain XPS spectra on ESCALAB 250Xi (Thermo Fisher Scientific Inc., USA). The diameter of the analyzed area was 400 μm.

#### ^1^H-NMR electrolyte analysis

For electrolyte extraction, the coin cells with different anodes were disassembled in a glove box. All the parts of the disassembled cell were then quickly transferred into a centrifugal tube containing 2 ml of diluent and shook for 5 min. After mixing for 2 or 24 hours, the electrolyte–dimethyl sulfoxide (DMSO) mixture was then extracted and analyzed using liquid NMR on a JNM-ECZ400S spectrometer. The internal standard substance is DCBF (Adamas, 98% purity), filling in the coaxial inner tube. The volume ratio of the DCBF and deuterated DMSO (d-DMSO) is 1:100. d-DMSO (99.9%, with 0.03% tetramethylsilane) was purchased from Anhui Zesheng Technology Co. Ltd.

### Electrochemical measurements

All coin cells were assembled with standard CR2025 coin-type cells in an Ar-filled glove box with O_2_ and H_2_O content below 0.1 ppm. The polypropylene film (Celgard 2400) was used as the separator. In different electrolyte systems, LiPF_6_ (98% purity), FEC (98% purity), EC (99% purity), DMC (99% purity), lithium bis(fluorosulfonyl)imide (LiFSI; 99.9% purity), DME (battery grade), 1,1,2,2-tetrafluoroethyl-2,2,3,3-tetrafluoropropylether (HFE; 99.9% purity), and lithium nitrate (LiNO_3_; 99.99% purity) were purchased from Suzhou Duoduo Chemical Technology Co. Ltd. The LHCE consists of LiFSI, DME, and HFE with a mole ratio of 1.0:1.8:2.0. A LiNO_3_ sustained-release membrane was used in the cell with LHCE. The fabrication and components of the LiNO_3_ sustained-release membrane have been described in a previous publication (*51*). The amount of electrolyte is 50 μl in the half and full coin cells.

The routine Li was fabricated by the molten method as described above to exclude the effect of the annealing and rolling process. The routine Li and Al-HCGB-Li were stamped into 14 mm in diameter for coin cells. The LiNi_0.5_Co_0.2_Mn_0.3_O_2_ (NCM523, 3.0 and 4.0 mAh cm^−2^) is used as a cathode in full cells. The NCM523 full coin cells were monitored in galvanostatic mode within a voltage range of 2.8 to 4.3 V. They were first cycled at 0.1 C and then cycled at 0.4 C. The charge/discharge tests of all coin cells were performed on a Neware BTS-51 multichannel battery tester (Shenzhen, Neware, BTS) in a testing room with a constant temperature of 25°C.

The pouch cells were assembled in a dry room with a dew point temperature of −50°C. A layer-by-layer process has been used to alternate the cathodes and anodes with polyethylene separators in pouch cells. All pouch cells were tested on a Land CT2001 multichannel battery tester (Wuhan Land Electronic Co. LTD.) in the environmental oven (Shanghai, Espec, Environmental Equipment Corp., SEG-021) with a constant temperature of 25°C and under a pressure device to provide a pressure of 1 MPa. The Al-HCGB-Li | NCM523 pouch cells were monitored within a voltage range of 3.0 to 4.3 V. They were first cycled at 0.05 C and then cycled at 0.1 C. The electrolyte of the pouch cell is 1.0 M LiPF_6_ in FEC and DMC (the volume ratio is 1:4).

### Computational method

First-principles calculations based on DFT were performed to reveal the interfacial effect on the reactivity of Li atoms using the Vienna Ab initio Simulation Package (*55*). The projector augmented wave method was used to describe the ion-electron interaction (*56*). Perdew-Burke-Ernzerhof version of generalized gradient approximation was adopted for the exchange-correlation energy (*57*). A kinetic energy cutoff of 320 eV was used for the plane wave expansion of the valence electron wave functions. A dense Γ-centered Monkhorst-Pack k-point mesh with a sampling density of 0.03 Å^−1^, 10^−6^ eV cell^−1^ in total energy, and 5 × 10^−2^ eV Å^−1^ in force was adopted for the convergence criterion during structural optimization. The site energy is defined as *E*_site_ = *E*_total_ − *E*_vacancy_ to evaluate the reactivity of Li atoms, in which *E*_total_ and *E*_vacancy_ are the total energy of a complete structure and a structure containing a vacancy, respectively. The deformation charge density is calculated by the difference between the total valence charge density of the structure and the superposition of the valence charge densities of neutral atoms.
