# Supplementary material for: Inhibiting intercrystalline reactions of anode with electrolytes for long-cycling lithium batteries
Source: Sci Adv. 2022 Aug 17;8(33):eabq3445. doi: 10.1126/sciadv.abq3445 (PMC9385152; doi:10.1126/sciadv.abq3445)
Supplement: Supplementary file 1 — Figs. S1 to S26 Tables S1 and S2 [file sciadv.abq3445_sm.pdf]

Science Advances
AAAS

Supplementary Materials for

Inhibiting intercrystalline reactions of anode with electrolytes for long-cycling lithium batteries

Peng Shi *et al.*

Corresponding author: Xue-Qiang Zhang, zhangxq@bit.edu.cn; Qiang Zhang, zhang-qiang@mails.tsinghua.edu.cn

Sci. Adv. **8**, eabq3445 (2022)
DOI: 10.1126/sciadv.abq3445

This PDF file includes:

Figs. S1 to S26
Tables S1 and S2

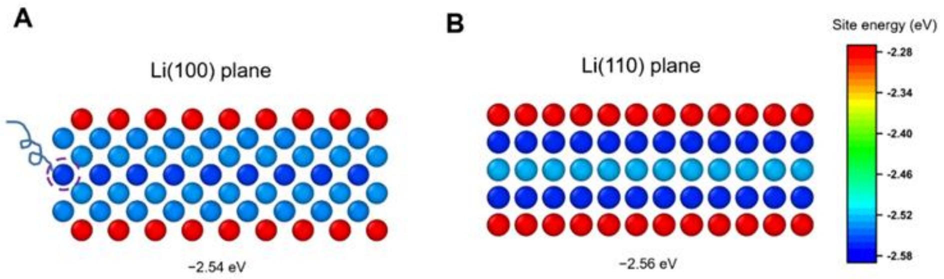

fig. S1. The site energy of Li atoms in (A) Li(100) and (B) Li(110) planes. The positive value (red color) and negative value (blue color) indicate an accumulation and depletion of the electron charge, respectively.

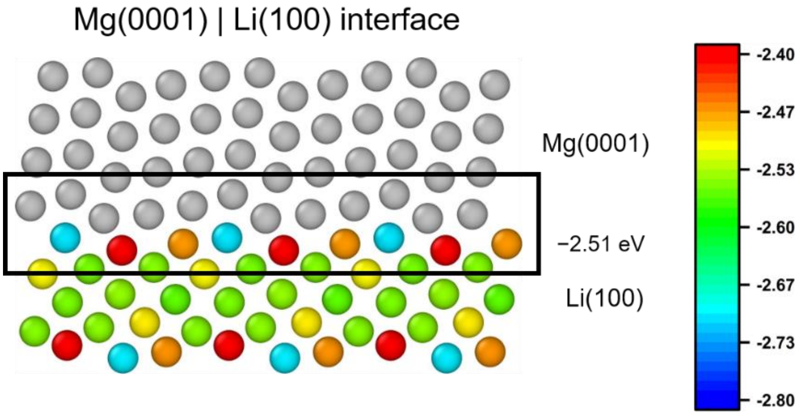

fig. S2. **Calculated site energy of Li atoms in Li(100) plane and the interface of Li and Mg metals.**

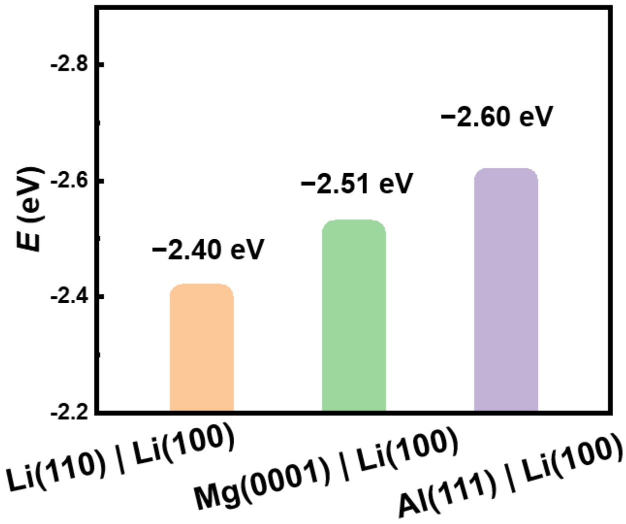

fig. S3. **Summary of the site energy of the Li atoms in different boundaries.**

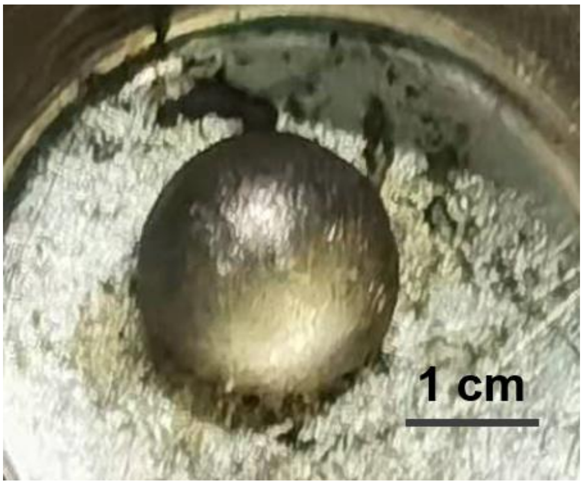

fig. S4. **The photo of the molten Al-HCGB-Li foil.**

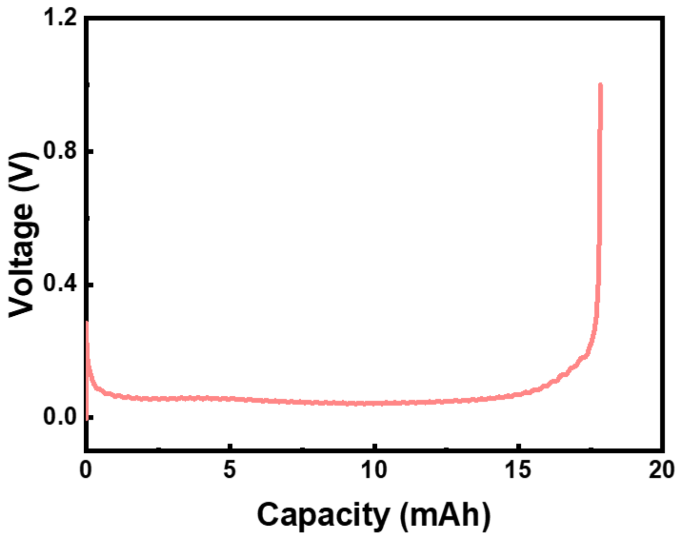

fig. S5. **Typical Li stripping curves of the Al-HCGB-Li anode at a current density of 0.5 mA cm^{-2} up to 1.0 V**. The capacity of the electrode is 17.83 mAh and the mass is 51 mg.

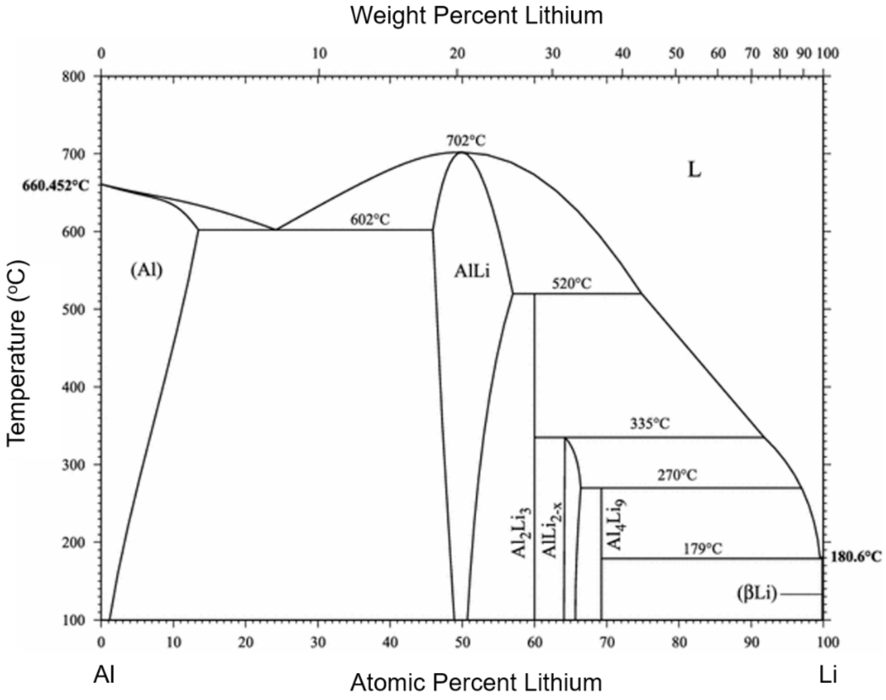

fig. S6. **Al-Li phase diagram.**

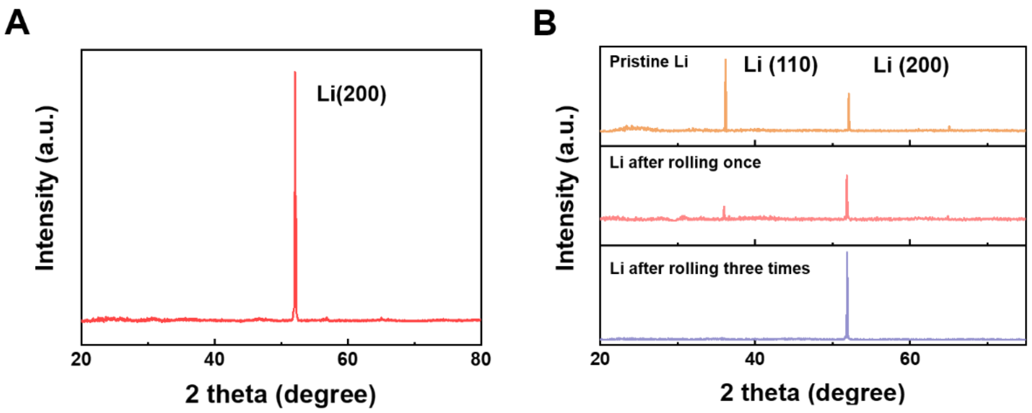

fig. S7. XRD pattern of the (A) Al-HCGB-Li foil and (B) pristine Li foil before and after rolling. The mechanical compression changes the texture of the metal, and thus the rolling process increases the proportion of exposed crystal planes of Li(200).

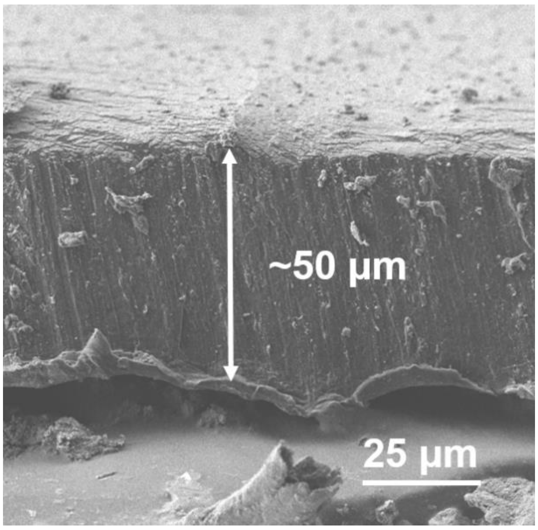

fig. S8. **The cross-sectional SEM image of the Al-HCGB-Li foil after rolling.**

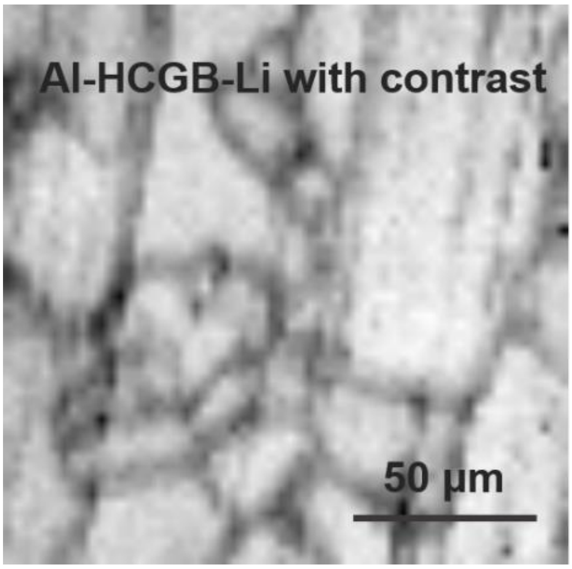

fig. S9. **The contrast map of the Al-HCGB-Li foil.**

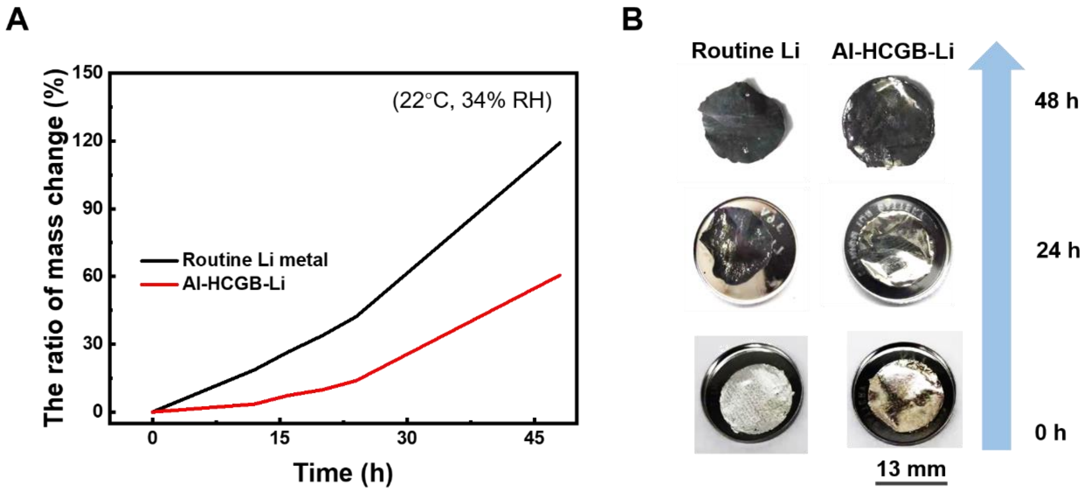

fig. S10. The air stability measurement of Al-HCGB-Li and routine Li metal. (A) The ratio of mass change and (B) morphology evolution of the Al-HCGB-Li and routine Li metal in the air with a humidity of 34%. The air stability measurement was conducted by placing the Al-HCGB-Li and routine Li metal in a stainless steel plate. The temperature and humidity were measured by a thermometer and hygrometer respectively.

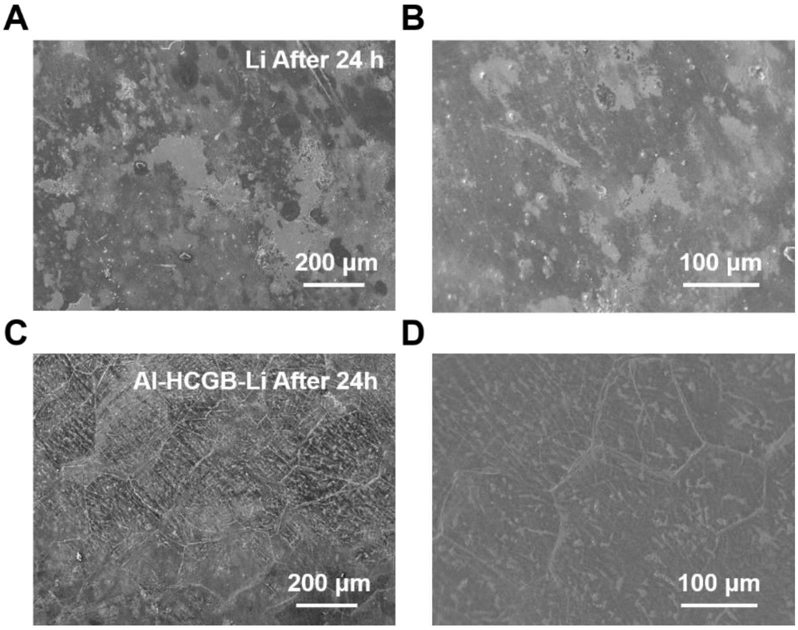

fig. S11. SEM images of the (A, B) routine Li metal and (C, D) Al-HCGB-Li foil soaked in the electrolyte of 1.0 M $LiPF_6$ in DMC. (B, D) is the partial enlargement of (A, C).

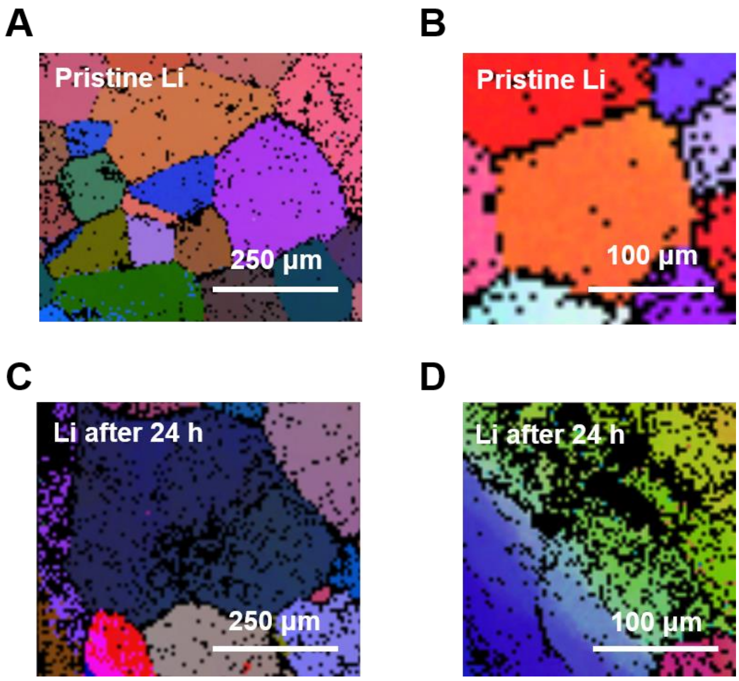

fig. S12. EBSD mapping of the routine Li metal (A, B) at the initial state and (C, D) soaked in electrolyte after 24 h.

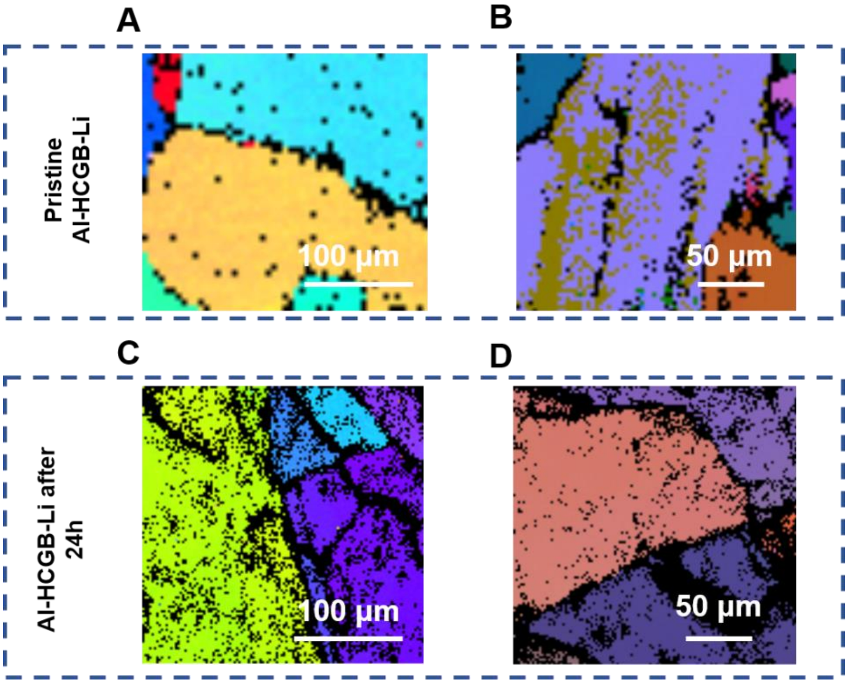

fig. S13. The EBSD mapping of the Al-HCGB-Li foil (A, B) at the initial state and (C, D) soaked in electrolyte after 24 h.

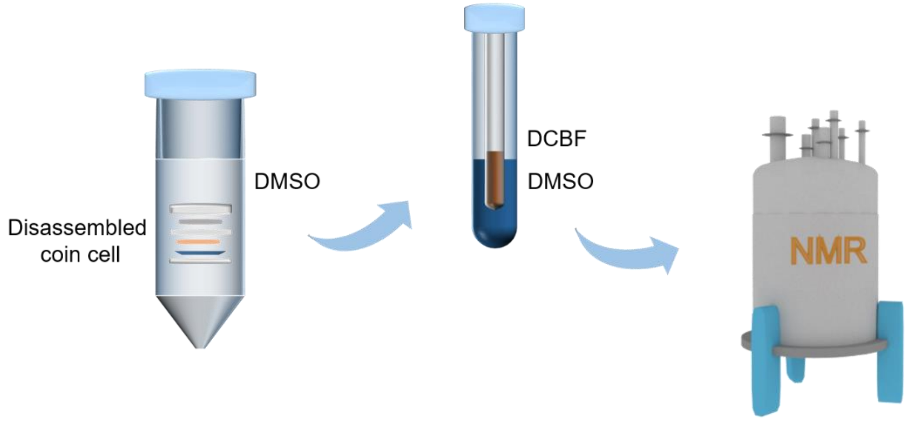

fig. S14. **Schematic of NMR experiments.** The disassembled coin cell was placed in a centrifugal tube with 2 mL of anhydrous d-DMSO. The electrolyte–DMSO mixture was then extracted and the internal standard substance of DCBF was added into the coaxial inner tube for quantification, and then it was analyzed employing liquid NMR. Moreover, the centrifugal tube with the disassembled coin cell was shelved for 24 h to magnify the effect of corrosion.

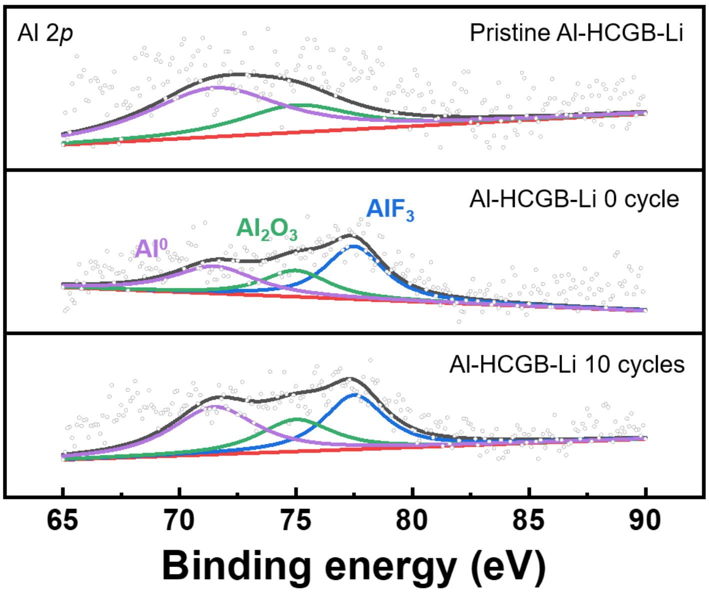

fig. S15. **XPS spectra of pristine Al-HCGB-Li, Al-HCGB-Li after 0 cycle and Al-HCGB-Li after 10 cycles.** The content of Al^0 and Al_2O_3 is 69.2% and 30.8% in the pristine Al-HCGB-Li, respectively. When the Al-HCGB-Li was assembled in the cells, a new peak of 77.2 eV is assigned to AlF_3 due to the reaction of Al and the electrolyte. The proportion of Al^0 and Al_2O_3 decreases to 41.3% and 23.2% in Al-HCGB-Li after 0 cycle, respectively, and the proportion of AlF_3 is 35.5%. After 10 cycles, the proportion of Al^0, Al_2O_3 and AlF_3 is 41.6%, 24.2% and 34.2%, respectively.

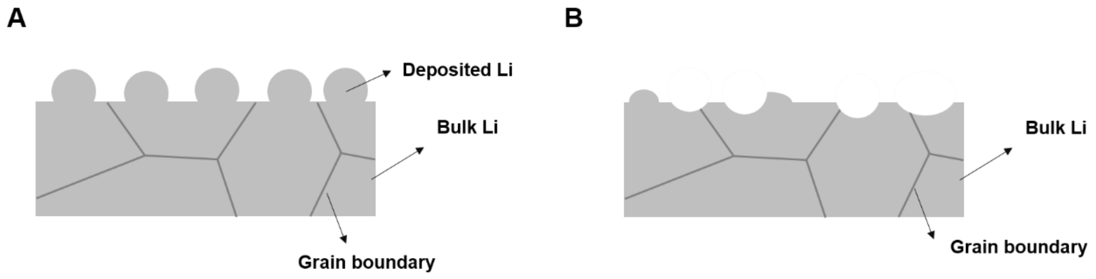

fig. S16. Schematic of deposited Li, bulk Li, and grain boundary during the process of (A) plating and (B) stripping.

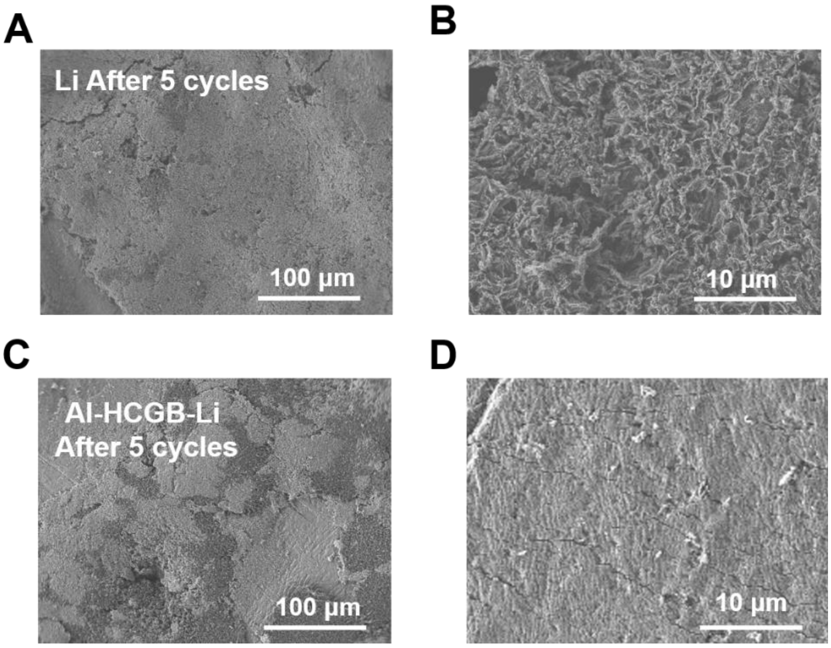

fig. S17. The morphology of (A, B) routine Li metal and (C, D) Al-HCGB-Li foil after 5 cycles in symmetrical cells. And the electrolyte is 1.0 M $LiPF_6$ in DMC. (B, D) is the partial enlargement of (A, C).

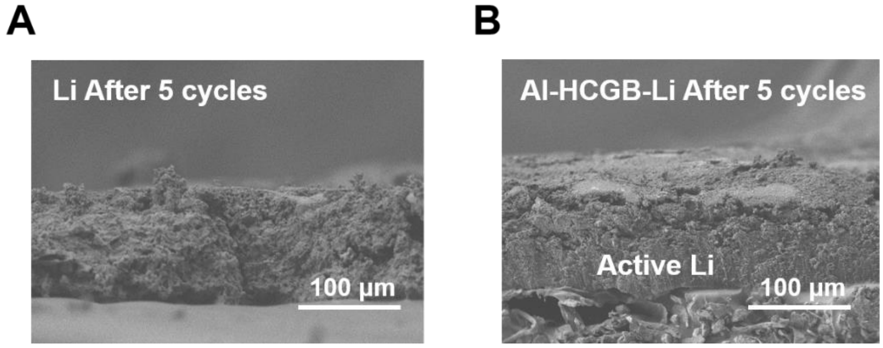

fig. S18. The cross-sectional morphology of (A) routine Li metal and (B) Al-HCGB-Li anode after 5 cycles in symmetrical cells. And the electrolyte of 1.0 M $LiPF_6$ in DMC.

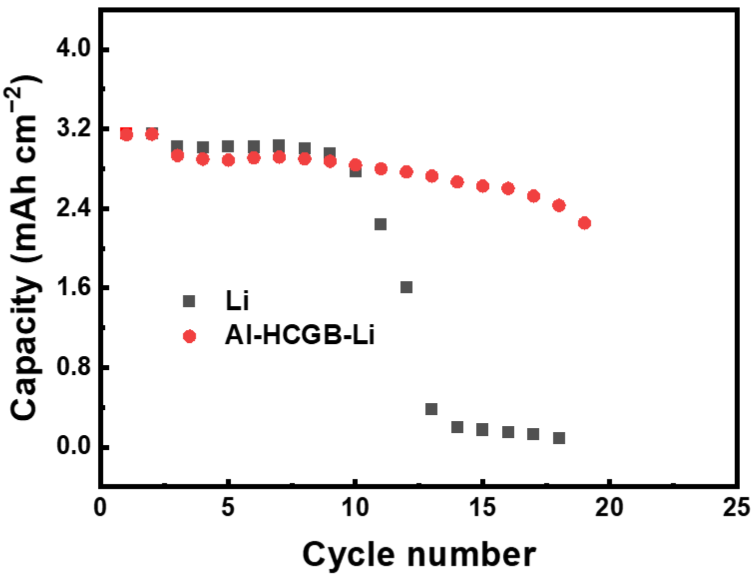

fig. S19. Cycling performance of the coin cell with routine Li metal and Al-HCGB-Li anode at 0.4 C. The electrolyte is 1.0 M $LiPF_6$ in DMC.

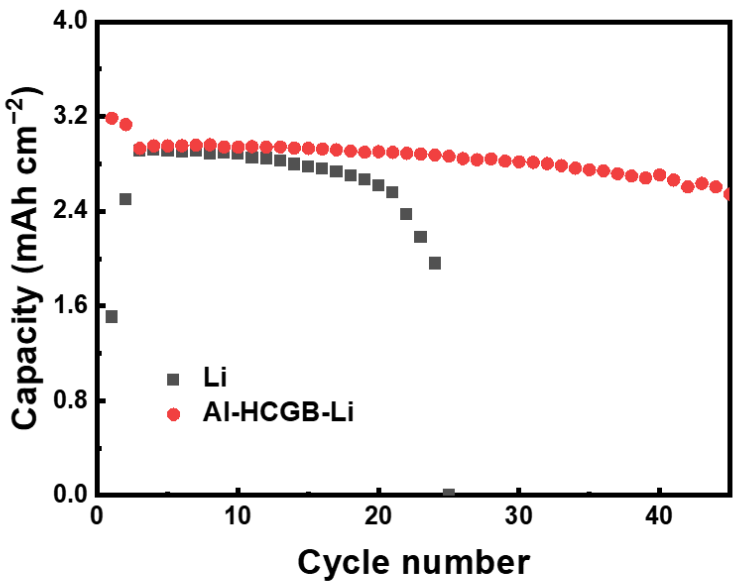

fig. S20. Cycle performance of the coin cell with routine Li metal and Al-HCGB-Li anode at 0.4 C. The electrolyte is 1.0 M $LiPF_6$ in EC/DMC (The volume ratio is 1:1).

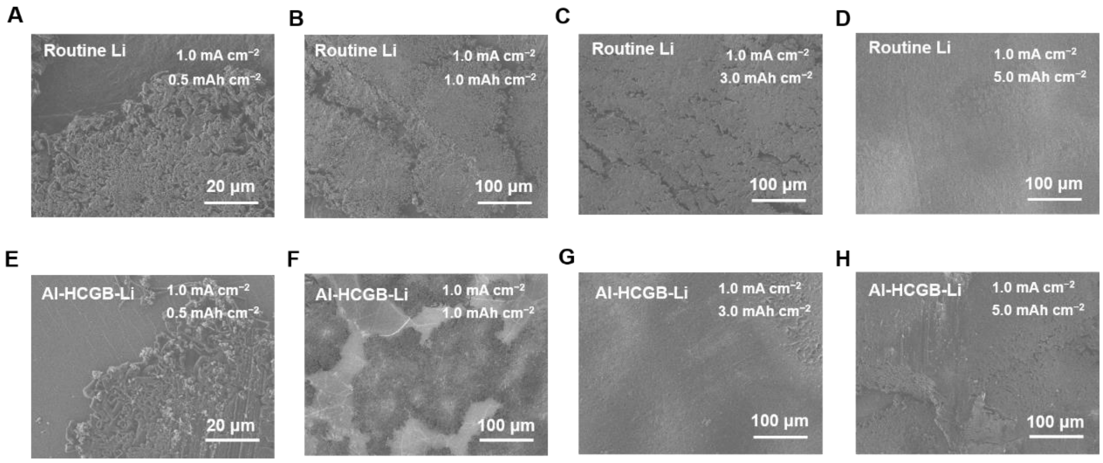

fig. S21. **The morphology of Li plating on (A–D) routine Li and (E–H) Al-HCGB-Li at 1.0 mAcm^{-2} and different capacities.** (A, E) 0.5 mAh cm^{-2}, (B, F) 1.0 mAh cm^{-2}, (C, G) 3.0 mAh cm^{-2} (D, H) 5.0 mAh cm^{-2}. The electrolyte is 1.0 M $LiPF_6$ in FEC/DMC (The volume ratio is 1:4).

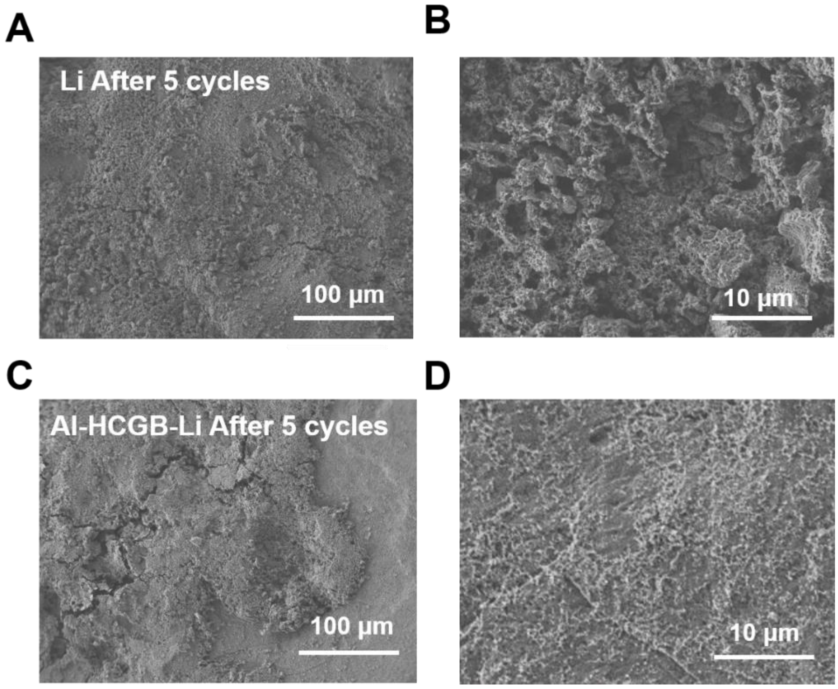

fig. S22. The morphology of (A, B) routine Li metal and (C, D) Al-HCGB-Li anode after 5 cycles at stripping state in full cells. The cathode is NCM523 and the electrolyte is 1.0 M $LiPF_6$ in FEC/DMC (The volume ratio is 1:4). (B, D) is the partial enlargement of (A, C).

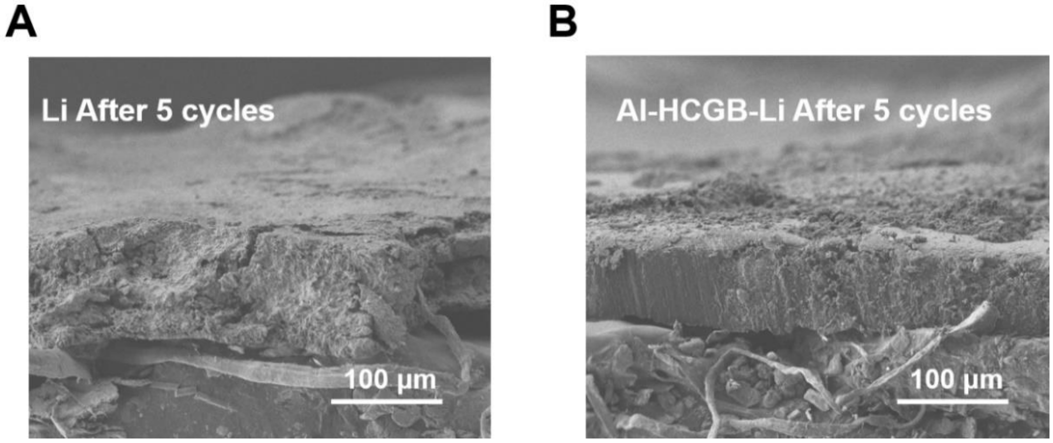

fig. S23. The cross-sectional morphology of (A) routine Li metal and (B) Al-HCGB-Li anode after 5 cycles in full cells. The cathode is NCM523 and the electrolyte is 1.0 M LiPF6 in FEC/DMC (The volume ratio is 1:4).

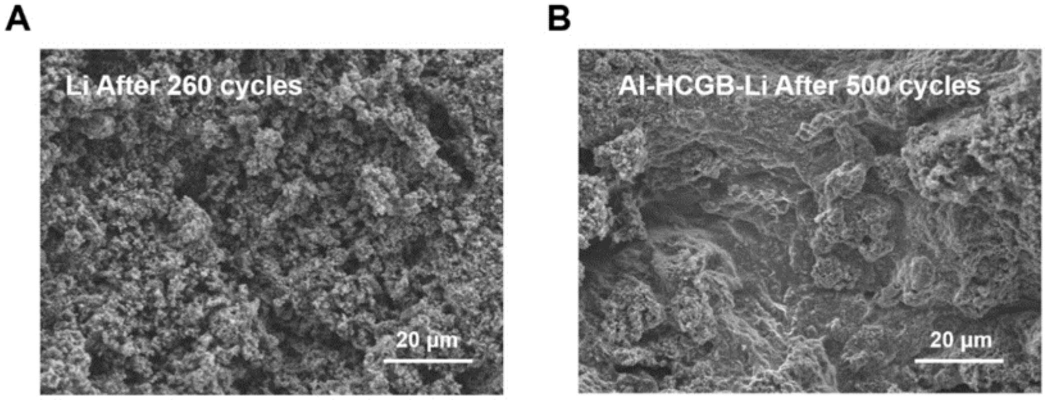

fig. S24. The morphology of (A) routine Li metal after 260 cycles and (B) Al-HCGB-Li anode after 500 cycles in full cells. The cathode is NCM523 and the electrolyte is LHCE with the $LiNO_3$ additive.

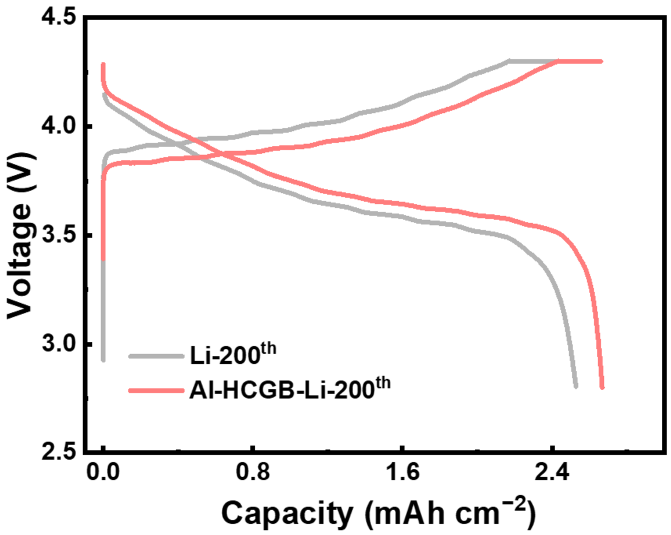

fig. S25. The voltage profiles of full cells at the 200th cycle in LHCE with the $LiNO_3$ additive.

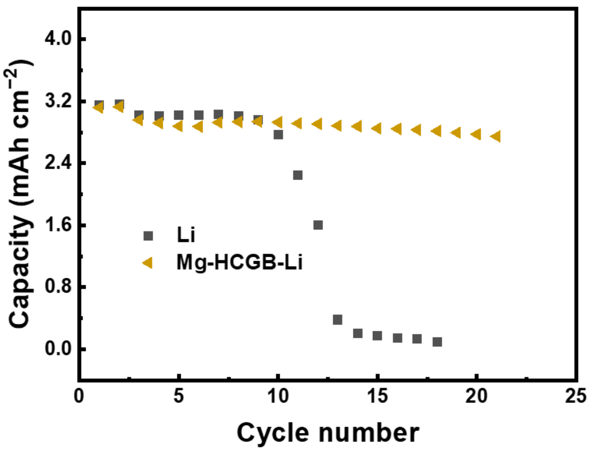

fig. S26. **Cycling performance of the coin cell with routine Li metal and Mg-HCGB-Li anode at 0.4 C.** The electrolyte is 1.0 M $LiPF_6$ in DMC.

table S1. Quantity changes of FEC and PF_6^- calculated according to NMR.

**Sample**	**FEC changes (%)**	PF_6^- **changes (%)**
Routine Li 0 cycles	0.00	0.00
Routine Li 10 cycles	71.39	71.02
Al-HCGB-Li 0 cycles	0.00	0.00
Al-HCGB-Li 10 cycles	25.50	33.14

table S2. The specifications of the Al-HCGB-Li | NCM523 pouch cell.

Cell component	Specification	Parameters
Cathode (NCM523)	Active material mass loading (mg cm^{-2}, each side)	25.1
	Active material content	0.96
	Number of electrodes	17
	*Specific areal capacity (mAh cm^{-2}, each side)	4.0
	Weight (g) (including Al collectors)	25.1
Anode (Al-HCGB-Li)	Number of electrodes	18
	Thickness (μm)	50
	Weight (g) (including Cu collectors)	4.09
Electrolyte	electrolyte/capacity (g Ah^{-1})	2.3
	Weight (g)	8.91
Separator (PE)	Weight (g)	1.14
package and lugs	Weight (g)	1.7
Full cell	Dimension (cm×cm)	7×4
	N/P	2.5
	Discharge capacity (Ah)	3.84
	Mid-value voltage (V)	3.78
	**Discharge energy (Wh)**	**14.52**
	**Total weight (g)**	**40.89**
	****Specific energy (Wh kg^{-1})**	**355.1**

* Specific areal capacity in this table is the measured value, not the theoretical value.

**Specific energy = discharge energy (Wh) / total weight (kg). This is a more precise calculation method compared to the method: Specific energy =discharge capacity (Ah) × mid-value voltage (V) / total weight (kg).
